# Supplementary material for: Contamination Status and Acute Dietary Exposure Assessment of Paralytic Shellfish Toxins in Shellfish in the Dalian Area of the Yellow-Bohai Sea, China
Source: Foods. 2024 Jan 23;13(3):361. doi: 10.3390/foods13030361 (PMC10855875; doi:10.3390/foods13030361)
Supplement: Supplementary file 1 [file foods-13-00361-s001.zip › foods-2720112-supplementary.pdf]

Table S1 Uncertainty analysis of acute dietary exposure to PST from shellfish in general consumers

Age groups	Statistics	P2.5	P25	P50	P75	P97.5	QR	95%CI
3~6	Mean	0.436	0.447	0.451	0.458	0.467	0.011	(0.436, 0.467)
	P50	0.239	0.244	0.247	0.248	0.254	0.005	(0.239, 0.254)
	P75	0.527	0.534	0.542	0.548	0.560	0.014	(0.527, 0.560)
	P90	0.989	1.021	1.034	1.050	1.069	0.028	(0.989, 1.069)
	P95	1.425	1.494	1.545	1.562	1.616	0.069	(1.425, 1.616)
	P97.5	2.011	2.094	2.156	2.192	2.365	0.098	(2.011, 2.365)
	P99	2.781	3.010	3.090	3.221	3.438	0.211	(2.781, 3.438)
	P99.9	4.974	6.377	6.726	7.080	8.625	0.702	(4.974, 8.625)
7~12	Mean	0.375	0.383	0.386	0.389	0.394	0.006	(0.375, 0.394)
	P50	0.219	0.222	0.224	0.225	0.231	0.003	(0.219, 0.231)
	P75	0.451	0.458	0.464	0.467	0.475	0.010	(0.451, 0.475)
	P90	0.828	0.849	0.860	0.868	0.882	0.019	(0.828, 0.882)
	P95	1.214	1.242	1.274	1.295	1.325	0.054	(1.214, 1.325)
	P97.5	1.635	1.726	1.762	1.794	1.924	0.068	(1.635, 1.924)
	P99	2.300	2.537	2.587	2.659	2.831	0.122	(2.300, 2.831)
	P99.9	4.312	4.790	5.074	5.629	6.162	0.838	(4.312, 6.162)
13~18	Mean	0.278	0.283	0.287	0.290	0.298	0.007	(0.278, 0.298)
	P50	0.166	0.170	0.172	0.174	0.177	0.004	(0.166, 0.177)
	P75	0.339	0.347	0.353	0.360	0.367	0.013	(0.339, 0.367)
	P90	0.611	0.631	0.642	0.651	0.668	0.020	(0.611, 0.668)
	P95	0.879	0.903	0.918	0.941	0.989	0.038	(0.879, 0.989)
	P97.5	1.195	1.229	1.274	1.312	1.381	0.083	(1.195, 1.381)
	P99	1.566	1.765	1.837	1.891	2.044	0.126	(1.566, 2.044)
	P99.9	2.800	3.249	3.423	3.924	4.438	0.674	(2.800, 4.438)
19~59	Mean	0.240	0.241	0.242	0.242	0.244	0.001	(0.240, 0.244)
	P50	0.137	0.138	0.138	0.139	0.139	0.001	(0.137, 0.139)
	P75	0.284	0.285	0.287	0.288	0.290	0.002	(0.284, 0.290)
	P90	0.535	0.538	0.540	0.541	0.545	0.003	(0.535, 0.545)
	P95	0.779	0.789	0.793	0.795	0.805	0.006	(0.779, 0.805)
	P97.5	1.094	1.108	1.116	1.122	1.139	0.014	(1.094, 1.139)
	P99	1.602	1.625	1.642	1.677	1.726	0.053	(1.602, 1.726)
	P99.9	3.275	3.434	3.520	3.613	3.750	0.180	(3.275, 3.750)
≧60	Mean	0.210	0.213	0.215	0.216	0.218	0.003	(0.210, 0.218)
	P50	0.123	0.124	0.125	0.126	0.127	0.002	(0.123, 0.127)
	P75	0.254	0.257	0.260	0.261	0.265	0.004	(0.254, 0.265)
	P90	0.468	0.474	0.478	0.482	0.490	0.008	(0.468, 0.490)
	P95	0.667	0.687	0.696	0.704	0.716	0.018	(0.667, 0.716)
	P97.5	0.900	0.930	0.937	0.957	0.989	0.027	(0.900, 0.989)
	P99	1.288	1.394	1.432	1.453	1.528	0.059	(1.288, 1.528)
	P99.9	2.642	2.885	3.024	3.148	3.392	0.263	(2.642, 3.392)
Total	Mean	0.262	0.263	0.264	0.265	0.266	0.001	(0.262, 0.266)
	P50	0.146	0.147	0.148	0.148	0.149	0.001	(0.146, 0.149)
	P75	0.308	0.309	0.310	0.311	0.313	0.002	(0.308, 0.313)

	P90	0.589	0.592	0.594	0.595	0.599	0.003	(0.589, 0.599)
	P95	0.866	0.869	0.873	0.878	0.885	0.009	(0.866, 0.885)
	P97.5	1.218	1.232	1.238	1.248	1.261	0.016	(1.218, 1.261)
	P99	1.802	1.835	1.855	1.869	1.922	0.034	(1.802, 1.922)
	P99.9	3.725	3.850	3.919	3.965	4.134	0.114	(3.725, 4.134)
